# Patient Partner Perspectives Regarding Ethically and Clinically Important Aspects of Trial Design in Pragmatic Cluster Randomized Trials for Hemodialysis

**DOI:** 10.1177/20543581211032818

**Published:** 2021-07-26

**Authors:** Stuart G. Nicholls, Kelly Carroll, Cory E. Goldstein, Jamie C. Brehaut, Charles Weijer, Merrick Zwarenstein, Stephanie Dixon, Jeremy M. Grimshaw, Amit X. Garg, Monica Taljaard

**Affiliations:** 1 Clinical Epidemiology Program, Ottawa Hospital Research Institute, ON, Canada; 2 Department of Philosophy, Western University, London, ON, Canada; 3 School of Epidemiology and Public Health, University of Ottawa, ON, Canada; 4 Department of Medicine, Western University, London, ON, Canada; 5 Department of Epidemiology and Biostatistics, Western University, London, ON, Canada; 6 Centre for Studies in Family Medicine, Western University, London, ON, Canada; 7 Department of Family Medicine, Western University, London, ON, Canada; 8 Schulich School of Medicine & Dentistry, Western University, London, ON, Canada; 9 ICES, Ontario, Canada; 10 Lawson Research Institute, London, ON, Canada; 11 Department of Medicine, University of Ottawa, ON, Canada; 12 Division of Nephrology, Department of Medicine, Western University, London, ON, Canada; 13 Nephrology, London Health Sciences Centre, ON, Canada

**Keywords:** pragmatic cluster randomized trial, research ethics, qualitative research, informed consent, gatekeeper, trust

## Abstract

**Background:**

Cluster randomized trials (CRTs) are trials in which intact groups such as hemodialysis centers or shifts are randomized to treatment or control arms. Pragmatic CRTs have been promoted as a promising trial design for nephrology research yet may also pose ethical challenges. While randomization occurs at the cluster level, the intervention and data collection may vary in a CRT, challenging the identification of research participants. Moreover, when a waiver of patient consent is granted by a research ethics committee, there is an open question as to whether and to what degree patients should be notified about ongoing research or be provided with a debrief regarding the nature and results of the trial upon completion. While empirical and conceptual research exploring ethical issues in pragmatic CRTs has begun to emerge, there has been limited discussion with patients, families, or caregivers of patients undergoing hemodialysis.

**Objective:**

To explore with patients and families with experience of hemodialysis research the challenges raised by different approaches to designing pragmatic CRTs in hemodialysis. Specifically, their perceptions of (1) the use of a waiver of consent, (2) notification processes and information provided to participants, and (3) any other concerns about cluster randomized designs in hemodialysis.

**Design:**

Focus group and interview discussions of hypothetical clinical trial designs.

**Setting:**

Focus groups and interviews were conducted in-person or via videoconference or telephone.

**Participants:**

Patient partners in hemodialysis research, defined as patients with personal experience of dialysis or a family member who had experience supporting a patient receiving hemodialysis, who have been actively involved in discussions to advise a research team on the design, conduct, or implementation of a hemodialysis trial.

**Methods:**

Participants were invited to participate in focus groups or individual discussions that were audio recorded with consent. Recorded interviews were transcribed verbatim prior to analysis. Transcripts were analyzed using a thematic analysis approach.

**Results:**

Two focus groups, three individual interviews, and one interview involving a patient and family member were conducted with 17 individuals between February 2019 and May 2020. Participants expressed support for approaches that emphasized patient choice. Disclosure of patient-relevant risks and information were key themes. Both consent and notification processes served to generate trust, but bypassing patient choice was perceived as undermining this trust. Participants did not dismiss the option of a waiver of consent. They were, however, more restrictive in their views about when a waiver of consent may be acceptable. Patient partners were skeptical of claims to impracticability based on costs or the time commitments for staff.

**Limitations:**

All participants were from Canada and had been involved in the design or conduct of a trial, limiting the degree to which results may be extrapolated.

**Conclusions:**

Given the preferences of participants to be afforded the opportunity to decide about trial participation, we argue that investigators should thoroughly investigate approaches that allow participants to make an informed choice regarding trial participation. In keeping with the preference for autonomous choice, there remains a need to further explore how consent approaches can be designed to facilitate clinical trial conduct while meeting their ethical requirements. Finally, further work is needed to define the limited circumstances in which waivers of consent are appropriate.

## Abstract (fr)

**Contexte:**

Les essais randomisés en grappes (CRT — *Cluster Randomized Trials*) sont des essais dans lesquels des groupes intacts, comme des centers ou horaires d’hémodialyse, sont répartis aléatoirement dans des groupes traités ou témoins. Les CRT pragmatiques ont été présentés comme un modèle prometteur pour la recherche en néphrologie, mais susceptible de poser des défis sur le plan éthique. Bien que la répartition aléatoire ait lieu au niveau du groupe, les interventions et la collecte de données peuvent varier dans un CRT, ce qui peut complexifier l’identification des participants. Aussi, lorsque le comité d’éthique de la recherche accorde une dérogation au consentement des patients, une question ouverte se pose quant à savoir si, et dans quelle mesure, les patients devraient être informés de la recherche en cours ou recevoir un compte rendu sur la nature et les résultats de l’étude une fois celle-ci terminée. Alors que des recherches empiriques et conceptuelles explorant les questions éthiques dans les CRT pragmatiques commencent à poindre, peu de discussions ont eu lieu avec les patients sous hémodialyse, leurs familles ou leurs soignants.

**Objectifs:**

Explorer les défis posés par différentes approches de conception des CRT pragmatiques en contexte d’hémodialyse avec des patients ayant de l’expérience en recherche sur l’hémodialyse et leurs familles. Plus précisément : connaître leur avis sur a) l’utilization d’une renonciation au consentement; b) les processus de notification et les renseignements fournis aux participants; et c) toute autre préoccupation concernant les CRT en contexte d’hémodialyse.

**Type d’étude:**

Entrevues et groupes de discussion sur la conception d’essais cliniques hypothétiques.

**Cadre:**

Les groupes de discussion et les entrevues ont eu lieu en personne, par vidéoconférence ou par téléphone.

**Participants:**

Les patients partenaires de recherche en hémodialyse — définis comme des patients ayant une expérience personnelle en dialyze ou un membre de leur famille avec de l’expérience dans l’accompagnement d’un patient en hémodialyse — qui ont participé activement à des discussions pour conseiller une équipe de recherche sur la conception, la conduite ou la mise en œuvre d’une étude en hémodialyse.

**Méthodologie:**

Les participants ont été invités à participer à des discussions individuelles et des groupes de discussion enregistrés avec leur consentement. Les enregistrements ont été transcrits intégralement avant l’analyze et les transcriptions ont été analysées en utilisant une approche d’analyze thématique.

**Résultats:**

Deux groupes de discussion, trois entrevues individuelles et une entrevue avec un patient et un membre de sa famille ont été menés auprès de 17 personnes entre février 2019 et mai 2020. Les participants ont exprimé leur appui aux approches qui privilégient le choix des patients. La divulgation des risques et des renseignements concernant le patient était un thème clé. Les processus de consentement et de notification ont tous deux généré de la confiance, mais le fait de contourner le choix du patient a été perçu comme une atteinte à celle-ci. Les participants n’ont pas écarté l’option d’une renonciation au consentement, mais ont été plus restrictifs quant au moment où celle-ci serait acceptable. Les patients partenaires se sont montrés sceptiques quant aux allégations d’impraticabilité fondées sur les coûts ou l’engagement en temps pour le personnel.

**Limites:**

Tous les participants étaient canadiens et avaient participé à la conception ou à la conduite d’un essai, ce qui limite le degré d’extrapolation des résultats.

**Conclusion:**

Puisque les participants préfèrent avoir le choix de participer à une étude, nous pensons que les chercheurs devraient étudier attentivement les approches qui permettent aux participants de faire un choix éclairé en cette matière. Conformément à la préférence pour un choix autonome, il demeure nécessaire d’explorer plus profondément la façon dont les approches de consentement peuvent être conçues pour faciliter la conduite des essais cliniques tout en respectant leurs exigences éthiques. D’autres travaux sont nécessaires pour définir les rares circonstances où une renonciation au consentement serait appropriée.

## Introduction

Studies have shown substantial variation in practice and patient outcomes across hemodialysis facilities.^1^ In part, this has been attributed to a lack of high-quality randomized controlled trials upon which to base practice.^2,3^ Accordingly, there have been calls for methodological innovation to address this deficiency.^4^

Pragmatic cluster randomized trials (CRTs), which randomize intact groups (eg, entire hemodialysis facilities or shifts) to different interventions can facilitate the evaluation of treatment protocols or strategies that are implemented at the institutional level.^5^ The randomization of whole facilities can also reduce logistical complexities as staff within facilities only need to administer a single intervention. Furthermore, pragmatic trials are intended to inform a clinical or health policy decision, and cluster randomization has also been promoted as a more pragmatic trial design that may be used in hemodialysis settings.^6,7^ For example, randomization at the cluster level may better reflect the clinical context of how treatments or policies are delivered in practice. Furthermore, when routinely collected data are available for outcome assessment, cluster randomization with a waiver of consent may facilitate the cost-efficient inclusion of whole clusters, potentially increasing generalizability.^8^

The hemodialysis setting is well suited for pragmatic CRTs^3^ with frequent and regular patient contact, clinically relevant data collected on a routine basis, and with standardized approaches to care for patients within each center. Consequently, the number of pragmatic CRTs conducted in nephrology has increased, and they potentially address some of the challenges to conducting trials in hemodialysis.^5^

Despite the potential benefits offered by pragmatic CRTs, they pose ethical challenges.^9^ While randomization occurs at the cluster level (eg, the hemodialysis facility), the units of randomization, intervention, and data collection may vary in a CRT, which poses challenges for the identification of research participants, and consequently questions about to whom research protections are owed. In a CRT, clusters are commonly allocated to the study interventions before research participants can be identified and recruited, and some interventions may be adopted as the facility protocol during the course of the trial. This raises the question of from whom, when, and for what consent should be obtained. Moreover, when a waiver of patient consent is granted by a research ethics committee, there is an open question as to whether and to what degree patients should be notified about ongoing research or be provided with a debrief regarding the nature and results of the trial upon completion. Furthermore, pragmatic designs that integrate research into routine care have the potential to blur which elements constitute research and those that constitute quality improvement or innovative practice. Consequently, some authors have queried what information needs to be disclosed to patients with respect to research-specific risks (in contrast to clinical risks).^10,11^

While empirical and conceptual research exploring ethical issues in pragmatic CRTs has begun to emerge,^9,12-16^ there has been limited discussion with patients undergoing hemodialysis and their families or other caregivers. This is despite the increasing popularity of CRTs and increasing interest in patient engagement in research.^17,18^ The aim of this study was to address this gap and explore the perceptions of patients and their families about ethical issues in pragmatic CRTs in the hemodialysis setting. Specific questions were as follows:

1. What ethical issues are raised by cluster randomization?
2. What are the perceived benefits of and concerns with using altered consent approaches, including a waiver of consent?
3. What are the perceived strengths and drawbacks of different notification processes and/or how information may be provided to participants?

## Methods

To address these questions, we used focus groups and semi-structured interviews to explore attitudes toward different scenarios reflecting trial design alternatives. The study reporting is consistent with the COREQ checklist,^19^ and which is included in the Supplementary Material S1.

### Participants

Individuals were eligible for the study if they were identified as a hemodialysis patient partner, that is, if they were either a patient with personal experience of dialysis or a family member with experience supporting a patient on hemodialysis and had been previously involved in discussions to advise a research team on the design, conduct, or implementation of a hemodialysis trial. The inclusion of patient partners in study teams conducting pragmatic CRTs reflects the underlying premise that trials that are more pragmatic should address outcomes and study questions relevant to patients and clinical practice. While previous studies of ethical issues raised by pragmatic trials have included patients and members of the public,^20,21^ few studies draw on the perspectives of patient partners who may have had previous exposure to considerations arising from alternative study design choices and their implications for research ethics. Patient partners previously involved in such discussions with research teams, as opposed to patients without research experience, were therefore included to ensure participants were familiar with the context and constraints of clinical research and thus, might have more informed opinions and increased ability to respond to specific research questions. This approach has been previously applied to study the challenges posed by pragmatic trials more generally.^14,22^ Potential participants were required to be fluent in English.

### Identification and Recruitment

Potentially eligible patient partners were identified from hemodialysis trials or other research projects in our clinical networks, publications of pragmatic CRTs, and funding announcements for hemodialysis trials. The principal investigators were approached via email and asked to forward a study invitation and consent form to any interested patient partners. In addition, an advertisement for the study was placed on KidneyLink (www.kidneylink.ca) and in the Can-SOLVE CKD^23^ newsletter. If the patient partner indicated a willingness to participate, a time for the interview or focus group was scheduled.

### Interview Guide and Processes

Focus groups were conducted in-person or by videoconference. Videoconferencing software was used due to travel restrictions imposed by the COVID-19 pandemic and which prevented an in-person focus group. Individual interviews were arranged if the respondent had difficulty attending a focus group and were conducted by telephone or videoconference. All individual interviews were conducted over the telephone or via videoconferencing due to the geographic dispersion of participants and logistical considerations.

Similar to Kraybill et al.,^11^ both the focus groups and individual interviews followed a structured approach, starting with a 20- to 30-minute overview of the ethics of research, the distinction between research and clinical care, and explanation of randomization, cluster randomization, and pragmatic trial design. This was followed by a description of a hypothetical study exploring whether increasing the concentration of magnesium in dialysate improves health outcomes for patients on hemodialysis. This hypothetical scenario was designed to be realistic, drawing on existing studies^2,24-26^ and design principles for pragmatic comparative effectiveness trials. Participants were told that in the hypothetical study, researchers want to study a magnesium intervention because observational studies suggest that patients undergoing dialysis and who naturally have higher magnesium levels in the blood have better health outcomes. Consequently, it was explained that researchers were interested in exploring whether increasing the levels of magnesium in the dialysate could achieve higher blood magnesium levels and, ultimately, better outcomes for patients. However, this had not been evaluated in a trial.

The hypothetical study compared two formulations of dialysate. One arm of the trial involved a higher level of magnesium in the dialysate (intervention arm). Patients in the other arm of the trial would receive the concentration currently in use at their hemodialysis facility which would be lower than in the intervention arm (usual care arm). Both levels were explained to be within the range of existing magnesium levels given to patients on hemodialysis, with the higher dialysate magnesium levels reflecting the upper threshold of what was currently being provided in some centers (see Supplementary Material S2 for more details).

After introducing the hypothetical study, five scenarios were described and discussed. The five scenarios corresponded to different ways in which the study could be designed. Scenarios varied the unit of randomization (individual versus cluster randomization), the approach to consent (ranging from full written informed consent to a waiver of consent), and the ability for individual patients to avoid exposure to the intervention (see Table 1 for an overview of the scenarios and Supplementary Material S2 for further details).^27^ Scenario one was an individual patient randomized trial with a standard written informed consent process. The consent process was explained to participants as involving a 12-page consent form that provided the relevant information and that after reading this, participants would be expected to provide a written consent. Scenario two was a CRT with a written informed consent process, with additional probing regarding the possibility of post-randomization consent. In this scenario, the randomization was at the cluster level, but treatment was described as an individual-level treatment (an individual-cluster trial)^28,29^ as opposed to a cluster-wide intervention that cannot be avoided. Probes to consider alternative approaches to informed consent were used. Specifically, we included probes to consider variants such as a shortened consent form or “integrated consent.”^30,31^ The following scenarios (three, four, and five) were all CRTs with no informed consent but differed with respect to notification strategies. Scenario three provided no notification or option to opt out (ie, waiver of consent).^32^ Scenarios four and five maintained the use of a waiver of consent but introduced posters as potential method of notification and an opt-out procedure respectively.^33^ In addition, we probed alternative forms of notification, such as letters or leaflets. Each scenario was discussed in turn before proceeding to the next scenario. The information sets and scenarios were reviewed by two patient partners prior to the study starts and were revised for clarity based on the comments received.

**Table 1.** Breakdown of Consent Approaches and Scenarios.

	Study design	Consent	Ability to avoid intervention	Notification
Scenario 1	Patient randomization	Full written (intervention and data collection)	Yes	Yes—informed consent
Scenario 2	Cluster randomization	Full written (intervention and data collection) (+PROBE: Short written consent after conversation with doctor) (+PROBE: Verbal consent only)	Yes	Yes—informed consent
Scenario 3	Cluster randomization	No patient recruitment or consent (ie, waiver of consent)	No (but doctor may change treatment for individual patients)	None—patients are unaware of the trial (+PROBE: Debrief after trial completion)
Scenario 4	Cluster randomization	No patient recruitment or consent (ie, waiver of consent)	No (but doctor may change treatment for individual patients)	Posters (+PROBE: Letters or verbal)
Scenario 5	Cluster randomization	No patient recruitment or consent (ie, waiver of consent)	Yes—patients can opt out of being exposed to interventions	Posters inform patients, includes opt out information (+PROBE: Letters or verbal)

### Data Collection

Following the presentation of each scenario, participants were asked to consider whether they found the trial approach acceptable and whether they would participate. These were used as a starting point to discuss perceived challenges presented by the trial design, and which were explored by two standard probes: What do you think are the good things about this trial? What concerns might you have about this trial? Participants were asked to consider these from a societal and personal perspective. They were also encouraged to compare issues between scenarios. The interviewer probed respondents on issues that arose from these main discussions to generate discussion about general issues and the influences on personal decision-making that would be important for investigators to consider.

All interviews were facilitated by one member of the team (S.G.N.), a senior research associate with formal training and experience in the conduct of qualitative research. The interviewer guided discussions and asked clarifying questions as required. Additional members of the team (C.E.G., C.W., K.C., and M.T.) were also provided opportunities to ask questions.

In all cases, interviews were audio-recorded with consent, transcribed verbatim by a professional transcription service, and imported into NVivo 11^34^ qualitative data analysis software for analysis. In addition, field notes were taken by a member of the team (C.E.G.). During the process of transcription, data were de-identified and interview participants assigned a unique participant ID. Participants were provided their transcript for validation purposes. No additional comments or edits were received, and analysis proceeded on the basis of the original transcripts.

The study was approved by the Ottawa Health Sciences—Research Ethics Board (Ref: 20180133-01H).

### Data Analysis

The orientation of the study was one of qualitative description,^35^ a low inference approach in which discussion points were described and coded without interpretation. This analysis focused on the responses to the presented scenarios to capture specific comments about the aspects varied. We also compared coding across scenarios to identify common themes. Analysis proceeded through a thematic analysis^36^ in which we coded text inductively, with codes compared within and across groups and scenarios. We subsequently collated two analyses—a within-case analysis of comments and broader analysis of themes that cut across all the cases. To enhance trustworthiness and credibility of the analyses, a codebook was maintained throughout the study, and all transcripts were coded by one extractor (S. G. N.) and verified by another (K.C.).^37^ All transcripts were discussed, and individual codes and themes were revised until consensus was achieved.

## Results

Participant demographics are provided in Table 2. Two focus groups (one with seven participants and another with five participants), three individual interviews, and one interview involving a patient and family member were conducted. Overall, 17 individuals participated in the study. Interviews took place between February 2019 and May 2020, with sessions lasting between 67 and 132 minutes, including the presentation. All participants had been involved as an advisor for a multicenter trial and all participants were from Canada.

**Table 2.** Participant Demographics.

	Focus group 1 (N = 7)	Focus group 2 (N = 5)	Interviews (N = 5)	Overall (N = 17)
Sex				
Male	4	4	3	11
Female	3	1	2	6
Experience				
Patient	5	5	4	14
Family member or caregiver	2	0	1	3

We present our findings below. First, we report descriptive findings on responses to the design features that we systematically varied within the scenarios, namely: (1) challenges raised by individual versus cluster randomization, (2) informed consent and issues surrounding the use of waiver of consent, and (3) strengths and drawbacks of different notification processes and information provision for participants. Second, we report two cross-cutting themes: (4) trust in the nephrologist, and (5) confusion regarding research-related limitations. Illustrative quotes for each of the identified themes are presented in the text, with additional examples in Table 3.

**Table 3.** Exemplar Quotes for Key Themes.

Theme	Example quotes
1. Challenges raised by individual versus cluster randomization	
1a. The benefits of cluster randomization	1.1 “I think less mistakes will be made [in a cluster design] if it just stays the one standard across the unit. And if it gets raised then that’s fine it’s just a new protocol this is what everybody gets. So I think it will be the purest way to get the information that’s needed.” LM16
1b. The disclosure of the intervention to which their facility has been allocated	1.2 “I’d be 100% behind this one. It would be for me and I don’t see any problem with it. [. . .] because you’re going to get what the centre tells you. You don’t have a choice. The centre didn’t have a choice so either you participate or you don’t, but you have that decision so I don’t have a problem with that at all.” LM08 1.3 “You tell them you’re going to be getting more; are they now looking for differences that they wouldn’t necessarily have thought? Are you looking for blood results versus the person’s perception of what it is? You’re getting magnesium. . . Oh well, now I’m cramping or maybe it wouldn’t have been that big a deal before now—you’re looking for faults. [. . .] So, oh my God, my heart’s racing: is this the magnesium? I want out!” LM04
1c. Gatekeepers in cluster trials	1.4 “One thought that I had, and I’m wondering if maybe others had some thoughts on this: in centres (this would specifically be around haemodialysis units, but places that have a patient committee or a patient/family advisory committee or something like that), if it might be something where the patients could have a designated person or something that would be involved in reviewing these applications for CRTs. So it’s not just the doctors that are deciding about participation in a particular trial, but the doctors and administrators and nurses but also some patients that reviewed the trial application on behalf of the whole group of patients to say this is something that we would be interested in or wouldn’t be.” LM11 1.5 “But I guess my comment would be: is the whole idea about who decides whether a centre is participating or not? Hopefully it’s kind of by committee rather than a Director or a Vice-President saying ‘okay I’m making the executive decision that we’re going to take part in it’.” LM10
2. Informed consent and issues surrounding the use of a waiver of consent	
2a. The content of information disclosed to potential participants	2. 1 “[. . .] So, for example if adding the magnesium could in any way physically affect you following your run. Maybe you get dizzy (I don’t know about anybody else), your blood pressure’s up and down after, the blood comes back when it wants to, and you can have dizzy spells and those types of things. If that could make it worse. So I wouldn’t have a problem participating but I would want to know that it there’s potential for side effects that I should look out for not as a ‘hey this isn’t working’ but ‘by the way as part of this study you should be aware that it’s possible after that you may get dizzy’. So if the effect is simply better dialysis — great, but if there’s potential for negative side effects after when—you are with your medical staff that’s one thing—but when you go home and I live alone so you should maybe avoid the stairs for 20 minutes after your run unless you’d like to find yourself at the bottom of them.” LM04 2.2. “[. . .] the doctor told me that there was a trial on this new drug, and they were very hopeful for helping reduce rejection on your kidney. And he said to me that there’s only a 25% chance of getting diabetes. And I’m like: You mean when I’m taking the drug it was 25% and when I stopped taking the drug it would revert? Well no, no you would have diabetes (laugh). It’s the perceived risk, right. You’re saying well yeah well, you’d probably reduce the chance of rejection however you’re going to get something else. So, it, it’s one of (laugh) one of these questions that are, the doctor seemed to think it was a reasonable thing. Myself as a dialysis patient said oh thank God, I’m not a diabetic; it was one of the worst things that I could ever think of doing. So, you know, it’s all part of what you think is a reasonable risk and what the patient thinks is a reasonable risk.” LM12
2b. Length and format of consent	2.3 “somebody’s going to look at that [12-page consent form] and be like: I’m not reading that, and they might disregard it. . . You get a single page with one paragraph on there, or whatever, two paragraphs. . . I might be a little bit more inclined to read something like that whereas a 12-page thing I probably wouldn’t read that.” LM02 2.4 “Well I would try to break that down into a couple of pages but if it would be easy for the doctor to sign off and say ‘yes’ I would get the doctor to read all the technical stuff and just kind of break it down for me and put it in little; let’s say break it down to at least 2-3 pages. Because the last time I was going to sign a document and I had to read, I think it was eight pages, I said Nah, and I didn’t want to do it! It was just too much to do just to say, yeah you can do it and not know what you’re being responsible for. And I know that some dialysis patients say that, well I’m not going to sign this — it’s too many pages . . .” LM17 2.5. “I guess the first thing that jumps off the screen at me here is the 12-page consent document. And I guess in terms of your questions I think that would be it might dissuade some people from participating just because any large document is probably not going to get read. And I think people will be afraid of the unknown. I’m guessing a 12-page consent would probably be pretty thick in legalese which I think would be off-putting for people as well. So I guess my only comment would be if the intent is to get a lot of uptake from the patients you’d want to find a way to scale back the consent form to the core things that the patient needs to know is happening.” LM11 2.6 “I’m not going to just take a doctor’s verbal verbiage because, you know, my mind’s not what it used to be when I was in my 20s (laugh) so I’d probably forget half of the stuff he told me.” LM14 2.7 “Reading a document for some people may, it may as well be gobbly-gook [. . .] They need to actually be able to understand what’s going on. So there needs to either be a person or a study representative who fully explains what the document means and sort of answers the questions that they would have because they don’t necessarily understand that 12-page consent form.” LM04 2.8 “I definitely don’t feel that a strictly verbal presentation is enough. Because it’s a lot of information and it’s hard to keep it all in your head, and also in the fact that people learn and understand different ways. Some people are better verbally and some people are better with the written word [. . .].” LM16
2c. Timing of consent	2.9 “[. . .]near the end [of their dialysis] people, the patients, are more tired too. So, maybe think of the timing of when you get consent because it’s highly dependent on people actually participating.” LM06
2d. Waiver of consent	2.11 “I think I kind of agree with what LM11’s just saying there that you read this, it [scenario 3, no consent] sounds wrong in that you’re kind of slipping something past patients, but in reality we’re kind of relying on the doctors to make these decisions all the time. And in fact, talking to a doctor about magnesium specifically I think there is no standard and there’s lots out there. So as long as you’re not using something, some level of magnesium that’s maybe never been tried before, you know, something that is reasonable and you’re not expecting any problems I think I wouldn’t be nearly as concerned. I don’t think any of us probably know the magnesium level of our dialysate today.” LM15 2.12 “Is it not true already that there is slight variations in what care is delivered without ever explaining it to the nurses, the techs, change of supplier for dialysate and a patient switches to a different machine that has a different temperature profile. [. . .] They get a better deal from [SUPPLIER] than they get from [SUPPLIER] at one point. Like this happens already and we don’t feel the need to get consent. Is that accepted? I mean to me it seems reasonable, but it happens.” LM03 2.13 “I don’t necessarily buy the time or money argument because I think that everyone in their everyday life has to make decisions about what they can do and can’t do. And I think researchers have to understand that when you’re researching another human being you have to set aside time and money for gaining consent.” LM11 2.14 “[. . .], I don’t think anything like that [no consent because it is impractical] would fly because you can make the adjustment to do individual trials. So, it’s just the lazy ways of doing a trial that you don’t want to spend any money and you don’t want to put the time and effort into it.” LM14 2.15 “I, I would, I would think that I don’t think that’s an excuse not to get consent is because it’s too costly.” LM10 2.16 “LM14: So, if I get this right that you’ve done this trial without telling us and now you’re going to tell us about the results afterwards? Interviewer: Yeah, yeah LM14: Nice (laugh) thank you (everyone laughing) Interviewer: (laugh) Do I detect sarcasm? LM14: Yeah I don’t know if I’d really thank you.”
3. Strengths and drawbacks of different notification processes and information provision for participants	
3a. Notification to promote transparency	3.1 “And so that’s why it’s nice and they do say we’re doing this trial, you know, you feel like they’re not putting something over on you or trying to hide something.” LM09
3b. Content of notification and conveyance of opt out	3.2 “I think that’s [putting up poster with an explicit opt out statement] going to initiate that for a lot of people as soon as they see oh I can opt out of this that’s it done right away without taking a second look.” LM02 3.3 “Moderator: Okay, okay. So, you mentioned about not making it too easy for people to, to opt out? LM09: Right Moderator: I mean would you see any particular approaches that might be reasonable ways there? LM09: Well I guess I think it’s always easier for people to say yes than no, so don’t ask them yes or no, you know, say this is what we’re doing. Again if you have any concerns this is who you can contact, but it takes effort then to make that contact so most people, you know, just out of ease I guess choose to do nothing.” 3.4 “If you wanted this to work you just can’t put a poster out there and let it sit, let’s say, 3-4 weeks, 5 weeks. I would put it in a timeframe [. . .] we’re doing that study, and this is when it will end. If we’re going to use that and if people want to opt out. I would say go with scenario 5 and just say there’s a timeframe here to opt out.” LM17 3.5 “Like you need to opt out, you know, please call within the next seven days to opt out, kind of thing. Because if not then, I can see it also would be easier like, well you said I had the chance to opt out and I’m like yeah but it’s three months from the time I told you. (laugh) Right like as humans we very easily put things off and then get mad at the outcome even though it was our own procrastination.” LM16
3c. Reach of information	3.6 “[. . .] put one by the scale but everybody has to go and weigh in and weigh out. That if they’re put at eye level, you know, I guess average height or whatever and then if you didn’t catch them when they were sitting there weighing in or weighing out but everybody has to either sit and wait to come in or wait to leave so put them in the waiting area. And then one on the door going in and then one as you’re coming out of the dialysis unit as you’re walking by into the hallway to leave, you know, so just coming and going and waiting.” LM08 3.7 “I think if you’re going to let them opt out I don’t think putting a poster up is the way to go. Because I, I think you just open yourself up later when you say well there was a poster up why didn’t you opt out? Not everyone reads everything, not everyone looks at the bulletin board or at the walls and reads all this stuff. So, I just think you’re setting yourself up to seem like you were trying to sneak something by them rather than, than giving them the real true option.” LM15 3.8 “I have no problem with posters whatsoever, but I think a combination of nephrologist, nurse, researcher conversation with them and the posters would work best.” LM08 3.9 “I think the posters are a good idea, but I think they should give patient individual letters as well. You know if you’re in a dialysis if you’ve ever been in a dialysis unit there’s so many posters in the waiting room that they get lost. And I think it gives the patient a sense of they care about you as an individual if they give you an individual letter.” LM09
4. Trust in the nephrologist	4.1 “Oh and then the last thing I thought of that 12-page consent form I don’t know about that (laugh) I go to the end and sign it and I just, you know, trust that whatever I was being told was right. I don’t know that I would sit there and read through 12 pages to get to the end.” LM08 4.2 “Well I’ve read consent forms and I admit I’ve signed a few without reading line by line [laughing] just on the basis, you know, the study partner or the nephrologist sort of having established trust. And you don’t know how many of these are written in plain language or are they written to be protection from legal repercussions? Is it really looking for informed consent or is it to forestall possible issues for them down the road.” LM03 4.3 “And I think a lot of times what it basically comes down to is trust. We trust our physicians. We trust those that are caring for us. When that trust is broken and, you know, and a way of breaking it would be to do these trials and not tell us, you know, then the outcomes are different. But, you know, we trust that what they’re telling us is correct.” LM09 4.4 “But I also trust very much like we have three nephrologists who rotate for two weeks we’ll have one doctor, two weeks we’ll have the next one and two weeks we’ll have the next one. And I am very comfortable with all three of them so I would be absolutely fine with any of them or all of them because they likely would all decide together, deciding yes we would like our unit to opt in.” LM16 4.5 “This one’s [Scenario 3] probably the best for the study [. . .] So as long as my doctor is aware that the study’s happening and notes any potential side effects or any ill effect of the study, then I mean if it’s reasonably safe or at least safe enough that the study is willing to do it and the hospital’s willing to do it then and it’s best for the study.” LM04
5. Confusion regarding research-related limitations	5.1 “We were talking about the individual recipe for dialysis for each of us and that is key. I know that when we go to different centres, they send the recipe along with us. And so I think I would be most comfortable if my doctor came along and said to me I think you would do better on a higher level of magnesium I’d like to try it for a few weeks to see how it works for you, what do you think? And with that reassurance from my doctor that in his professional opinion, or her, was going to be for the positive I would probably jump at it quite eagerly. And I know that’s not pure research with large numbers of people but that’s a situation where I would be a comfortable research subject because my doctor thought that there was a chance of some real benefit here.” LM12 5.2 “Yeah I like this one better [Scenario #3]. I’d rather have the doctors decide because they know a little bit more what’s happening with your body. And then they can pick and choose like the people like let’s say because I know there’s a lot of people that have heart conditions or diabetes too and some have cancer while they’re here. Yeah, I think a doctor would be the best to decide for the patients.” LM17 5.3 “And if there’s harm coming to you, the doctor’s going to adjust your level, but yeah, this is the way it is in the real world so I don’t have a problem with it at all.” LM08

*Note.* CRTs = cluster randomized trials.

### Responses to Design Features

#### 1. Challenges raised by individual versus cluster randomization

The first design feature of interest was patient randomization versus cluster randomization. Participants discussed three main topics: (1a) the benefits of cluster randomization, (1b) the disclosure of the allocated intervention, and (1c) the identification of gatekeepers.

##### 1a. The benefits of cluster randomization

Comments concerning the change from individual to cluster randomization mainly related to the perceived logistical simplicity, rather than scientific, or ethical implications of the design. For example, participants discussed the increasing simplicity for running the trial or minimizing the burden to the center staff (Quote 1.1).

##### 1b. The disclosure of the intervention to which their facility has been allocated

When probed regarding the potential for disclosure of the intervention to which their facility had been allocated, participants varied in their response, but none indicated a strong desire to know whether their center had been randomized to the higher dialysate level or to usual care. Some participants indicated that they would participate in the trial irrespective of the intervention to which their cluster had been randomized (Quote 1.2). One reason for this view was that participation would not result in a lower quality of care compared to their current care:

I don’t know if I would want to know [which group they were in] necessarily [. . .] you’re not going to be any worse off no matter which arm you’re in. You’re really getting your regular care or you’re going to get something stepped up. So yeah, I guess that’s fine — I’m good either way to tell you the truth. It would not matter to me whether I knew ahead of time or not. *LM08*

Participants did, however, indicate a desire to know the purpose of the trial and alternatives to participation. Other reasons for withholding information about the intervention allocation were related to trial feasibility: some participants may refuse to participate if they discovered that their cluster had been allocated to the usual care arm.

I could understand as a patient being less inclined to want to take part [in the trial if they know they are in the usual care arm] or to be envious of the dialysis units that are ‘in,’ and insist that I want the higher protocol. *LM16*

This demonstrates an underlying assumption that the intervention (fixed higher level of magnesium) would be better than the existing care. In addition, participants suggested that knowledge about being in the intervention arm may lead to a tendency to ascribe any change in the patient’s health to the study intervention. This, it was suggested, could increase withdrawal from the study or reporting of side effects that may not otherwise have been reported (Quote 1.3).

##### 1c. The identification of gatekeepers

Others raised the question of gatekeeping. Gatekeepers are individuals or groups who may be called upon to protect the interests of cluster members. Researchers often approach gatekeepers—such as hemodialysis center directors—to permit the enrollment of their cluster in a trial. Participants commonly expected nephrologists to be involved in decisions regarding their center’s participation in a trial, but also identified a range of other potential gatekeepers who may be relevant to such a decision. These included administrators, nurses, and patient partners (Quote 1.4).

Notably, participants indicated that cluster-level gatekeeping decisions should not be made by single individuals and that a range of perspectives should be incorporated, including the possibility of forming a committee (Quote 1.5).

#### 2. Informed consent and issues surrounding the use of a waiver of consent

Across the variations in consent approaches, we identified several topics related to consent: (2a) information disclosed to potential participants; (2b) length and format of the consent; (2c) timing of consent; and (2d) acceptability of a waiver of consent.

##### 2a. The information disclosed to potential participants

While we probed regarding disclosure of the intervention to which their facility had been allocated (1b above), participants discussed the disclosure of other information. Time and again participants discussed the “real life” implications of trial participation, such as the time commitments or patient-borne burdens, and how this shaped their attitudes or experiences. Participants discussed that consent materials should provide patients with an understanding of the risks and potential side effects, not just in terms of immediate medical issues, but also how the trial might impact their day-to-day activities (Quote 2.1). As one participant noted:

But again, it’s part of the process and we sort of say my quality of life for these 5 hours is worth the quality after. Where I would be concerned is if the study could potentially have side effects. So, for example, if adding the magnesium could in any way physically affect you following your run. [. . .] I wouldn’t have a problem participating but I would want to know if there’s potential for side effects that I should look out for. Not as a “hey this isn’t working” but “by the way — as part of this study you should be aware that it’s possible after that you may get dizzy [. . .] so you should maybe avoid the stairs for 20 minutes after your run unless you’d like to find yourself at the bottom of them.” *LM04*

Participants also wanted to know how long the trial would take, potential time frames for side effects, and whether the levels of magnesium were within existing ranges or experimental. Participants mentioned that risks that may seem reasonable to a medical professional may not be reasonable from a patient perspective (Quote 2.2).

##### 2b. Length and format of consent document

A near universal comment was that long consent forms, such as the 12-page consent form proposed within the scenarios, have limited utility. Participants indicated that patients may not read the form but sign it (Quotes 2.3) or that the mere presentation of a long form could serve as a potential barrier to participation (Quotes 2.4-2.5). These concerns over length were coupled with concerns over jargon: an assumption being that long forms would be full of “legalese.”

Despite concerns regarding jargon and the opacity of overly long consent forms, there was disagreement regarding the most appropriate approach to consent. While some participants indicated a verbal consent approach may be sufficient, several participants indicated that they would still wish to have a written consent approach. Written materials provided documentation that could be referred to later should one need to recall information, something that would be lost under different approaches (Quote 2.6).

Written consent materials were viewed as particularly valuable in trials of long duration as participants could review provided documents as needed. However, others noted that while written information is important in the context of disclosure, there was also an expectation that this would be discussed and the discussion would allow for clarification, such that written materials alone were not sufficient (Quotes 2.7-2.8).

Comments thus indicated that a one-size-fits-all approach would not suit everyone and that approaches that could adapt to different information needs based on literacy, inquisitiveness, and their state of mind at the time of approach, would be beneficial.

##### 2c. Timing of consent

A further aspect of discussion was the timing of the consent. Participants noted that approaching patients prior to starting dialysis was preferable to approaches made during or after a dialysis treatment session and where patients may be tired (Quote 2.9).

##### 2d. Waiver of consent

Reactions to the use of a waiver of consent varied but were informed by the intervention being studied. Some participants commented that their concerns about the use of a waiver of consent were allayed by the fact that the intervention arm used a level of magnesium concentration that was already being used in clinical practice. Similarly, the usual care arm and discretion of the physician to withdraw the patient also minimized their concerns (Quote 2.11).

This led to a discussion about when a waiver of consent is justified. Participants were less concerned about the use of a waiver for trials evaluating a center’s policy or aspects of care upon which patients usually have little or no input, such as purchasing dialysis machines (Quote 2.12). However, interventions involving direct interaction with the patient—such as an injection—were less accepted for use with a waiver of consent, as one participant noted:

So again, if we’re talking about the magnesium, there’s no way I would like this trial [without consent]. I’d rather a trial that I consent to. Now if the trial is about some sort of process or something like that that doesn’t actually go into my body then I’d be fine with it. *LM13*

When conditions of infeasibility were raised, participants roundly dismissed financial justifications (ie, costly to gain consent) or concerns about how long consent would take insofar as the trial involved non-urgent interventions (Quotes 2.13-2.15).

The practicality of maintaining a blinded status under a waiver of consent was also discussed: patients described their tendency to closely monitor their own data and felt that they would notice if the routinely monitored levels change. This would likely trigger a conversation with the nephrologist whereby disclosure of the trial would be clinically and ethically required.

One aspect that elicited a strong, and generally negative, response was the potential to debrief trial participants who had been engaged in a trial where no consent had been sought and where no notification was provided (Scenario three). Patient partners indicated that such a debrief would generate friction and likely decrease trust (Quote 2.16).

Notably, the length of the trial appeared to play a role for some patient partners with respect to how accommodating they were of a waiver of consent and debrief. Participants were more likely to accept a waiver of consent if the trial was of short duration. In such cases, trial results would soon be available to be shared with patients. In part, this appeared to reflect a broader perspective about acting on beneficial results and translating these into practice. However, some participants suggested that broad-scale information release after the conduct of a trial, such as information about its purpose, general information about the trial interventions, and results, could be more acceptable than a patient-specific debrief.

#### 3. Strengths and drawbacks of different notification processes and information provision for participants

The changes from Scenarios three to five, and inclusion of notification practices, raised three discussion topics: (3a) transparency, (3b) notification and opt-out, and (3c) reach of information.

##### 3a. Notification to promote transparency

Using posters to notify patients about ongoing research was perceived as transparent and honest (Quote 3.1). With respect to the posters themselves, two main aspects were discussed: their content and reach.

##### 3b. Content of notification

Participants mainly discussed whether the possibility to opt out should be on the notification materials. Some participants were concerned that notifying patients of the possibility to opt out would impact the scientific validity of the study but also noted the ethical need to inform patients. There was also discussion about people who would immediately opt out without considering the trial details (Quote 3.2). A suggestion was to have an opt-out mechanism, but to not make it overly simple (Quote 3.3).

A further issue highlighted by participants related to the practicalities of managing the opt-out process, including the provision of clear information to patients about this possibility (Quote 3.4-3.5).

##### 3c. Reach of information

With respect to the placement of posters, patient partners suggested several locations, such as on doors at the entrance to the hemodialysis center or above weigh scales (Quote 3.6).

However, some participants were concerned about the use of posters because some patients may not read English or have poor vision. This could be compounded if placed in certain locations, such as weighing scales, where participants may be focusing on their balance—in the context of frail patients—or may be focused on the scale results:

Often people with high falls risk aren’t concentrating on reading posters — they’re concentrating on being able to sustain balance while they’re in this precarious position. They may not read or write English; their vision may be bad. A lot of us are diabetic as well. You need to have robust ways for sharing that information whether it’s something taped to their dialysis machine or on their float, their run chart, or just you need to think beyond that simple poster. I think you would miss a lot of people. *LM03*

A second concern was due to “signage overload” that exists within medical centers. This was particularly salient within Scenario five, where the poster was the main conduit for informing patients. Others felt that notification through posters alone was too passive (Quote 3.7).

Participants supported other notification options, such as a letter or pamphlet given to all patients at the center, as a more active notification process (i.e., information is given directly to patients) rather than the passive use of posters. Yet, they emphasized that patient needs will vary and a plurality of approaches to notification would be beneficial (Quotes 3.8-3.9).

### Cross-Cutting Themes

#### 4. Trust in the nephrologist

Participants reported a high degree of trust in their nephrologist. When participants discussed not fully reading a consent form, trust in the nephrologist was a reason given (Quotes 4.1-4.2). This extended to others within the unit, which one participant described as being like a family:

Well you see them if you go every day. . . You build a little family when you go to dialysis especially when you’ve been on long-term. You see the regular people so if they see a regular face coming and you can explain it properly then it might be a lot easier to sell that way. *LM02*

However, this trust was earned through displays of honesty and transparency and could be endangered by not telling participants about trials in which they were enrolled (Quote 4.3). Patient partners also trusted their nephrologist to act in their individual best interests when enrolled in the trial (Quotes 4.4-4.5). Consequently, trust in the nephrologist was important both with respect to their role as a gatekeeper when enrolling a center in the trial but also for recruiting individual patients.

#### 5. Confusion regarding research-related limitations

Comments across the scenarios indicated that participants did not always understand the limitations that study participation places on their nephrologist. Specifically, comments reflected potential misunderstanding of the physician’s ability to act in the best interests of their individual care within the context of the trial. For example, some comments indicated a belief that their nephrologist could provide input with respect to which arm would be better for them, and this would inform their decision to take part (Quote 5.1).

When discussing the nephrologists’ role, patient partners supported their ability to withdraw patients from the trial. However, on occasions, this perceived research role appeared to be blurred with their clinical role (Quotes 5.2). As one participant noted:

And if there’s harm coming to you the doctor’s going to adjust your level, but yeah, I mean this is the way it is in the real world, so I don’t have a problem with it at all. *LM08*

There thus appeared to be the potential for therapeutic misconception or a blurring of research and practice and the nephrologist’s role within these areas.

## Discussion

Our study sought to explore patient partner views on ethical issues in pragmatic CRTs conducted in hemodialysis facilities. Few previous studies have empirically explored patient perspectives on alternative approaches to trial designs and their ethical implications.

A key message from this work was the importance of choice regarding trial participation. This is consistent with work by Courtright et al.,^38^ which found that participants were significantly more willing to participate in trials that employed a choice-based approach compared to trials that did not. Decisional autonomy appeared to be more important than the specific process for enabling their choice. Indeed, this reflects the distinction between the ethically grounded aspect of recognizing the patients’ autonomy and right to self-determination and institutionally formalized processes for documenting such decisions, and for which legal liability may be a motivating concern.^39,40^ Furthermore, and consistent with Weinfurt et al.,^41^ participants supported approaches that included active disclosure processes (such as leaflets given directly to patients) over more passive approaches, such as poster notification.

Participants articulated a need for patient-centered information within the consent process. They wanted to not only be told about clinical risks, but also how the trial may affect their daily activities. These findings correspond with previous workshops with patients that found that a major barrier to research was wariness about the potential burden of trial participation.^42^

Importantly, participants felt that the provision of information—through consent processes or notification—promoted transparency, illustrated respect for participants, and was a key element to establishing a trusting relationship.^43^ Participants noted that they have a pre-existing trust relationship with their nephrologist built up through their clinical interactions, but this may be undermined by withholding information about ongoing research. This reflects findings from workshops in Australia^42^ and focus groups in the United States,^11^ both of which identified trust in the clinician as a key component to patient decision-making.

Patient partners were amenable to alternatives to the standard long form written consent model but acknowledged that preferred formats and approaches may differ between patients. Simplified consent approaches may thus be acceptable to patients undergoing hemodialysis. The enforced pivot to alternative consent approaches generated by the COVID-19 pandemic has highlighted the availability of electronic consent (e-consent) approaches which have been adopted at scale and can be more broadly applied post-pandemic. A recent review of e-consent studies found that participants undergoing e-consent tended to have both subjectively and objectively better comprehension compared to paper consent forms and that there may be cost savings to study teams.^44^ Despite this, several authors have noted challenges regarding inequities of access and understanding of electronic options and that an e-consent approach may not be suitable for all participants.^45,46^ The e-consent approach is also being adopted in the hemodialysis research. The ongoing HiLo trial, for example, uses tablet devices to access secure web-based electronic consent modules, with the hope to obtain consent from over 4000 patients.^47^ In this regard, the experiences of studies in the hemodialysis context will be informative from both participant and researcher perspectives.

Despite renewed attention toward alternative approaches to informed consent within pragmatic randomized trials,^20,31,32^ discussion of consent approaches has tended to focus on the use of single uniform approaches. The feasibility of consent approaches that are adaptable to patient preferences remains under-explored.

While choice was preferred, participants did not dismiss the option of a waiver of consent. They were, however, restrictive in their views about when a waiver of consent may be acceptable. One key aspect relevant to the application of a waiver of consent is whether it would be impracticable to carry out the research if informed consent were required.^48^ A particular consideration is the relative impact that consent may have on cost, with some authors arguing that when obtaining consent imposes prohibitively high costs then consent may be deemed impracticable.^49^ This is particularly relevant in the present context as pragmatic trials that are publicly funded—and which may have smaller per-participant budgets—may be at greater risk of “resource-dependent impracticability.”^27,50^ Patient partners in this study were, however, skeptical of claims to impracticability based on costs or the time commitments for staff. The acceptability of different claims to impracticability remains an area in need of clarification and existing guidance is jurisdiction specific.^9^ Indeed, a recent review of international policy frameworks suggested that the limited use of altered and waived consent approaches in some jurisdictions “may, in part, be due to the absence of clear, trial-specific policy.”^51^

A further consideration raised by participants was the roles of gatekeepers, and the potential role patients can play as gatekeepers. The role of gatekeepers in the conduct of CRTs was addressed in the *Ottawa Statement on the Ethical Design and Conduct of Cluster Randomized Trials*^52^ although it does not specifically discuss the roles and responsibilities of diverse stakeholders in pragmatic trials. Recent work has suggested that an important ethical feature of more pragmatic trials is that they may involve a broader range of stakeholders than trials that are more explanatory in nature.^53^ The suggested potential for patients to serve as gatekeepers is consistent with the support for greater engagement and involvement of patients, families, and caregivers in hemodialysis research,^54-56^ and the creation of initiatives, such as Can-SOVE CKD,^57^ that include patient perspectives within hemodialysis research and care. While the engagement of patients, families, and carers has brought an increased focus to patient priorities, important questions remain regarding the specific responsibilities of different gatekeepers in dialysis care.^58^

Finally, there is a need to examine the role of debriefing in the context of trials conducted with a waiver of consent. Under Canadian ethics guidance, if a waiver of consent is granted, debriefing must be conducted “whenever it is possible, practicable and appropriate” (TCPS2, Article 3.7B).^59^ Even if researchers seek an exemption, they must provide a plan to disseminate information about the study to participants or their communities. Participants in our study were negatively disposed to the idea of a personal debrief in the absence of prospective informed consent, a result that was also found by Weinfurt et al.^41^ The lack of support for the debrief approach advocated in current guidance points to a need to engage patients in the development of ethics guidance. Further work remains to determine whether and how participants in pragmatic CRTs must be debriefed if enrolled under a waiver of consent.^60^

## Limitations

These results must be considered within the limitations of the study. First, our sample of patient partners is small and was solely drawn from Canada. Furthermore, we limited our sample to patient partners who had been involved in the design or conduct of a trial. While this was done to ensure familiarity with study design issues, and specifically experiences that could be drawn upon (as opposed to hypothetical or “off-the-cuff” responses), these opinions may differ from those of patients who have not been engaged in research design processes. There is the potential that the views expressed here—due to exposure to researcher perspectives—may be more in line with those of researchers than the general patient population. Thus we cannot make claims that our findings are generalizable to the broader population of patients treated with hemodialysis. Despite this, our results are largely consistent with other work in this space, and in other jurisdictions.^11^

Second, the structured nature of the scenarios and discussions likely means that we did not identify the full range of issues that may be pertinent to pragmatic CRTs in hemodialysis. Moreover, while we obtained patient feedback during the development of the scenarios to ensure clarity, the complex range of issues and scenarios may have been challenging to maintain at hand.

Third, the potential misconceptions regarding physician autonomy and best interests may have stemmed from information within the scenarios which emphasized that the nephrologist could withdraw the patient from the trial. While we cannot rule the misperceptions out as an artifact of the study scenarios, we again highlight the similarity in findings with previous work not only in hemodialysis,^11^ but across clinical trials more generally.^61,62^

## Conclusion

Patient partners supported approaches that allow patients to make an individual decision regarding trial participation. While choice was preferred, participants did not dismiss the option of a waiver of consent. They were, however, restrictive in their views about when such an approach may be acceptable. Patient partners were open to a range of alternative consent models, such as shortened consent forms with verbal disclosure and opt-out mechanisms. This should provide support to investigators that the choice is not a binary one between standard written informed consent or a waiver of consent and that an option that supports patient choice while also being feasible can be found. Moreover, patient input in the design of the trial may help identify consent options that can support patient autonomy, facilitate efficient and effective recruitment, and build trust through a transparent approach to trial conduct.

Despite the clear patient preference for decisional autonomy, there remains a need to further explore how alternative consent models can be designed to facilitate clinical trial conduct. This includes ongoing consideration as to when alternatives to standard written consent, including a waiver of consent, are ethically justifiable. Furthering this work will inform regulatory decision-making and assist investigators in their design choices.

## Supplemental Material

sj-pdf-1-cjk-10.1177_20543581211032818 – Supplemental material for Patient Partner Perspectives Regarding Ethically and Clinically Important Aspects of Trial Design in Pragmatic Cluster Randomized Trials for Hemodialysis Click here for additional data file. Supplemental material, sj-pdf-1-cjk-10.1177_20543581211032818 for Patient Partner Perspectives Regarding Ethically and Clinically Important Aspects of Trial Design in Pragmatic Cluster Randomized Trials for Hemodialysis by Stuart G. Nicholls, Kelly Carroll, Cory E. Goldstein, Jamie C. Brehaut, Charles Weijer, Merrick Zwarenstein, Stephanie Dixon, Jeremy M. Grimshaw, Amit X. Garg and Monica Taljaard in Canadian Journal of Kidney Health and Disease
