# Supplementary material for: Patient Partner Perspectives Regarding Ethically and Clinically Important Aspects of Trial Design in Pragmatic Cluster Randomized Trials for Hemodialysis
Source: Can J Kidney Health Dis. 2021 Jul 26;8:20543581211032818. doi: 10.1177/20543581211032818 (PMC8317238; doi:10.1177/20543581211032818)
Supplement: sj-pdf-1-cjk-10.1177_20543581211032818 – Supplemental material for Patient Partner Perspectives Regarding Ethically and Clinically Important Aspects of Trial Design in Pragmatic Cluster Randomized Trials for Hemodialysis [file sj-pdf-1-cjk-10.1177_20543581211032818.pdf]

COREQ (COnsolidated criteria for REporting Qualitative research) Checklist

A checklist of items that should be included in reports of qualitative research. You must report the page number in your manuscript where you consider each of the items listed in this checklist. If you have not included this information, either revise your manuscript accordingly before submitting or note N/A.

Topic	Item No.	Guide Questions/Description	Reported on Page No.
**Domain 1: Research team and reflexivity**			
Personal characteristics			
Interviewer/facilitator	1	Which author/s conducted the interview or focus group?<br>**SGN conducted the groups**	Page 11
Credentials	2	What were the researcher's credentials? E.g. PhD, MD<br>**PhD – see author details**	Page 11
Occupation	3	What was their occupation at the time of the study?<br>**Senior Research Associate**	Page 11
Gender	4	Was the researcher male or female?<br>**Male**	Not reported
Experience and training	5	What experience or training did the researcher have?<br>**formal training and experience in the conduct of qualitative research**	Page 11
Relationship with participants			
Relationship established	6	Was a relationship established prior to study commencement?<br>**No**	Not reported
Participant knowledge of the interviewer	7	What did the participants know about the researcher? e.g. personal goals, reasons for doing the research<br>**Participants were provided with background as to why the study was of interest as well as a 20- to 30-minute overview of the ethics of research, the distinction between research and clinical care, and explanation of randomisation, cluster randomisation, and pragmatic trial design.**	Page 8
Interviewer characteristics	8	What characteristics were reported about the inter viewer/facilitator? e.g. Bias, assumptions, reasons and interests in the research topic	Not reported
**Domain 2: Study design**			
Theoretical framework			
Methodological orientation and Theory	9	What methodological orientation was stated to underpin the study? e.g. grounded theory, discourse analysis, ethnography, phenomenology, content analysis<br>**Qualitative description was the orientation**	Page 12
Participant selection			
Sampling	10	How were participants selected? e.g. purposive, convenience, consecutive, snowball<br>**Purposive sampling was used**	Page 7-8
Method of approach	11	How were participants approached? e.g. face-to-face, telephone, mail, email<br>**By email**	Page 7-8
Sample size	12	How many participants were in the study?<br>**17**	Page 12

Non-participation	13	How many people refused to participate or dropped out? Reasons?<br>**N/A**	Page 12
Setting			
Setting of data collection	14	Where was the data collected? e.g. home, clinic, workplace<br>**Neutral venue, telephone or videoconference**	Page 8
Presence of nonparticipants	15	Was anyone else present besides the participants and researchers?<br>**Yes, other researchers**	Page 11
Description of sample	16	What are the important characteristics of the sample? e.g. demographic data, date<br>**Table 2**	Page 12, Table 2
Data collection			
Interview guide	17	Were questions, prompts, guides provided by the authors? Was it pilot tested?<br>**The information sets and scenarios were reviewed by two patient partners prior to study start and revised for clarity based on the comments received.**	Page 10
Repeat interviews	18	Were repeat inter views carried out? If yes, how many?<br>**No**	Not reported as N/A
Audio/visual recording	19	Did the research use audio or visual recording to collect the data?<br>**Audio recording**	Page 11
Field notes	20	Were field notes made during and/or after the inter view or focus group?<br>**Yes**	Page 11
Duration	21	What was the duration of the inter views or focus group?<br>**Between 67 and 132 minutes**	Page 12
Data saturation	22	Was data saturation discussed?<br>**no**	Page 11-12
Transcripts returned	23	Were transcripts returned to participants for comment and/or	Page 11
**Topic**	**Item No.**	**Guide Questions/Description**	**Reported on Page No.**
		correction?<br>**Yes**	
**Domain 3: analysis and findings**			
Data analysis			
Number of data coders	24	How many data coders coded the data?<br>**2 coders**	Page 12
Description of the coding tree	25	Did authors provide a description of the coding tree?<br>**Not reported – we provide an overview of the process**	-
Derivation of themes	26	Were themes identified in advance or derived from the data?<br>**Inductively coded**	Page 12
Software	27	What software, if applicable, was used to manage the data?<br>**NVivo 11**	Page 11
Participant checking	28	Did participants provide feedback on the findings?<br>**No**	-
Reporting			
Quotations presented	29	Were participant quotations presented to illustrate the themes/findings? Was each quotation identified? e.g. participant number<br>**Yes**	Table 3 and throughout results
Data and findings consistent	30	Was there consistency between the data presented and the findings?<br>**Yes**	Yes

Clarity of major themes	31	Were major themes clearly presented in the findings?	Yes
Clarity of minor themes	32	Is there a description of diverse cases or discussion of minor themes? **Yes, throughout the results we report on divergent opinions**	Pages 13-23

Developed from: Tong A, Sainsbury P, Craig J. Consolidated criteria for reporting qualitative research (COREQ): a 32-item checklist for interviews and focus groups. *International Journal for Quality in Health Care*. 2007. Volume 19, Number 6: pp. 349 – 357
